# Implementation Science to Respond to the COVID-19 Pandemic

**DOI:** 10.3389/fpubh.2020.00462

**Published:** 2020-09-02

**Authors:** Arianna Rubin Means, Anjuli D. Wagner, Eli Kern, Laura P. Newman, Bryan J. Weiner

**Affiliations:** ^1^Department of Global Health, University of Washington, Seattle, WA, United States; ^2^Public Health—Seattle and King County, Seattle, WA, United States; ^3^Office of Communicable Disease Epidemiology, Washington State Department of Health, Seattle, WA, United States; ^4^Department of Health Services, University of Washington, Seattle, WA, United States

**Keywords:** implementation science, COVID-19, public health response, novel coronavirus, SARS-CoV-2

## Abstract

The COVID-19 pandemic continues to expand globally, requiring massive public health responses from national and local governments. These bodies have taken heterogeneous approaches to their responses, including when and how to introduce and enforce evidence-based interventions—such as social distancing, hand-washing, personal protective equipment (PPE), and testing. In this commentary, we reflect on opportunities for implementation science to contribute meaningfully to the COVID-19 pandemic response. We reflect backwards on missed opportunities in emergency preparedness planning, using the example of PPE stockpiling and supply management; this planning could have been strengthened through process mapping with consensus-building, microplanning with simulation, and stakeholder engagement. We propose current opportunities for action, focusing on enhancing the adoption, fidelity, and sustainment of hand washing and social distancing; we can combine qualitative data, policy analysis, and dissemination science to inform agile and rapid adjustment to social marketing strategies to enhance their penetration. We look to future opportunities to enhance the integration of new evidence in decision-making, focusing on serologic and virologic testing systems; we can leverage simulation and other systems engineering modeling to identify ideal system structures. Finally, we discuss the ways in which the COVID-19 pandemic challenges implementation science to become more rapid, rigorous, and nimble in its approach, and integrate with public health practice. In summary, we articulate the ways in which implementation science can inform, and be informed by, the COVID-19 pandemic, looking backwards, proposing actions for the moment, and approaches for the future.

## Contributions to the Literature

- The COVID-19 pandemic response has illuminated gaps in emergency preparedness planning that could be addressed by implementation scientists working in coordination with public health practitioners.
- Classical implementation science tools and measures can be operationalized within the context of the COVID-19 pandemic, and at each stage of the pandemic. Doing so empowers implementation scientists to apply their skillsets toward optimizing uptake of evidence-based interventions such as such as social distancing, hand washing, and personal protective equipment.
- To be more responsive to public health emergencies, implementation science as a discipline must evolve to be more rapid, nimble, and policy-oriented in its approaches.

## Introduction

COVID-19 has emerged in early 2020 as a pandemic, marked by a classical exponential growth curve and global spread. National and local governments have taken heterogeneous approaches to curbing disease spread, relying on staggered and incomplete implementation of evidence-based public health behavioral prevention interventions, such as hand-washing and social distancing. State and local public health response efforts have focused on testing populations of public health significance, case and contact investigation, and infection prevention. Medical response to this novel virus has relied on limited supplies of personal protective equipment (PPE) for providers, ventilation assistance for severe cases, and testing. Development and trials of new interventions—including chemoprophylaxis and treatments, vaccines, and serologic tests—are underway.

Implementation science (IS) can be used to expand effective uptake of evidence-based interventions, such as hand washing, PPE use, ventilation, and social distancing. While epidemiology is the foundation of a robust pandemic response, IS methods can be used to understand how to protect historically excluded populations, rapidly engender necessary behavior change, and communicate data back to decision makers in a format that is actionable. IS relies on a broad toolkit from multiple disciplines including social science and mixed-methods, and is a necessary complement to traditional epidemiology. Domestically, IS has drawn heavily on frameworks, methods, and tools from behavioral science; in the global health context, IS draws on a broader array of frameworks, methods and tools (1). At the core of IS is the pursuit of effective and equitable collaborations and balancing systematization with system agility. In this Perspective piece, we articulate ways in which IS can inform, and be informed by, the COVID-19 pandemic.

## Operationalizing Is for COVID-19

IS focuses on characterizing and enhancing the acceptability, adoption, appropriateness, feasibility, fidelity, cost, penetration, and sustainability of evidence-based interventions in distinct settings (2). Within the context of COVID-19, we propose operational definitions of these outcomes at the individual, provider (clinical providers and public health workers broadly), and organizational levels, identify determinants (barriers and facilitators) to implementation, and distal clinical outcomes (Table 1). We further highlight current and anticipated areas of IS inquiry, relevant implementation outcomes, pragmatic methods, and implementation strategies that may be primed to activate targeted outcomes (Table 2) (26). Finally, we describe how the lessons from the COVID-19 pandemic can inform the discipline of IS to become a more nimble, rapid, and agile (27). In the sections that follow, we highlight specific selected examples in the text; however, Tables 1,2 provide rich and comprehensive examinations of the application of IS to COVID-19.

**Table 1:** Operationalizing Proctor implementation outcomes for COVID-19.

**Implementation outcome**	**Proctor definition**	**Level of operationalization**	**Specific application to COVID-19**	**Determinants**	**Distal clinical outcomes**
Acceptability	Perception among implementation stakeholders that a given treatment, service, practice, or innovation is agreeable, palatable, or satisfactory	Individual consumer Individual provider	Public agreement and personal values aligning with social distancing policies Health worker satisfaction with new hospital PPE guidelines	Race, class, and ability to social distance Management support, effective communication with health workers	Public trust and uptake of policies, community transmission reduction Perceived safety and health worker burnout
Adoption	Intention, initial decision, or action to try or employ an innovation or evidence-based practice	Individual provider Organization or setting	Attempted use of national testing guidelines City decision to adopt a regional policy of “shelter in place” or “stay home, stay healthy”	Clarity of guidelines, test kits available Acceptability of behavior changes to community members	Identification of positive cases and linkage to clinical management Community transmission reduction
Appropriateness	Perceived fit, relevance, or compatibility of the innovation or evidence based practice for a given practice setting, provider, or consumer; and/or perceived fit of the innovation to address a particular issue or problem	Individual consumer Individual provider Organization or setting	Compatibility between mask wearing and public life Perceived compatibility of PPE guidelines with actual need given hospital census and local epidemiology Perceived compatibility between existing and new pandemic-specific infrastructure, including supervisory systems and material distribution for COVID-19 response	Stigma associated with mask use, clarity of government guidance Trajectory of local outbreak, management support, effective communication with health workers Leadership knowledge of existing functioning systems prior to the outbreak	Community transmission reduction Perceived safety and health worker burnout Depending on type of materials being distributed, has implications for community transmission reduction, testing coverage, and subsequent positive case management
Feasibility	Extent to which a new treatment, or an innovation, can be successfully used or carried out within a given agency or setting	Individual provider Organization or setting	Extent to which health workers can accurately apply rapidly evolving virologic testing guidelines Degree to which hospital infrastructure can accommodate surges in patients requiring intensive care	Clarity of guidelines, effective management support, test kit, and PPE resource availability Number of staff available and allowed to perform intensive care, hospital beds and ventilators, availability of resources	Efficient test kit allocation to maximize identification of cases and link to clinical management, ability for health workers to return to work to perform clinical management for patients Reducing morbidity and mortality associated with severe cases
Fidelity	Degree to which an intervention was implemented as it was prescribed in the original protocol or as it was intended by the program developers	Individual provider	Public health practitioners implement supply chain for test kits, specimens, and case follow-up activities as designed	Availability of necessary resources, appropriate alignment between tasks and tasked personnel, effective communication that accounts for practitioner input	Reduction in community transmission
Implementation cost	The cost impact of an implementation effort	Provider or providing institution	Change in resource use at health facilities associated with social distancing	Political and economic pressure not to introduce “stay at home” restrictions	Increased resources for acutely ill (ex. ventilators), and reduction in morbidity and mortality
Penetration	Integration of a practice within a service setting and its subsystems	Organization or setting	Integrating processes (such as microplanning) for allocating resources across institutions and government offices, resulting in a harmonized approach to pandemic preparedness with multi-level consensus regarding leadership, communication, and management structures to be activated in an emergency	Leadership engagement, formal interorganizational networks, new resources made available for pandemic preparedness	Reduced community transmission, reduced hospital transmission
Sustainability	Extent to which a newly implemented treatment is maintained or institutionalized within a service setting's ongoing, stable operations	Administrators Organization or setting	Degree to which hospital leadership and government leadership do or do not maintain free coronavirus testing policies after the peak of the epidemic Degree to which innovative resource allocation modeling tools are institutionalized in public health and health facility planning practices, during stable operations	Coordination between policymaker and scientists, resource availability Learning climate, organizational culture, and political directives	Risk of future case surges Risk of future case surges

**Table 2:** Key implementation questions, outcomes, methods, and strategies for evidence-based COVID-19 interventions, at different stages of the pandemic.

	**Evidence-based intervention for COVID-19 control**	**Implementation question**	**Implementation outcome in question**	**Methods for addressing question**	**Implementation strategies with promise to address question**
Reflecting backward: opportunities for future emergency preparedness process/systems improvement	PPE	How can systems for stockpiling and subsequently distributing PPE be more automated and less reactive?	Sustainability	Process mapping (3, 4), stakeholder engagement (5), microplanning (6–8)	• Purposefully re-examine implementation (e.g., understand current processes and adjust as necessary) • Model and simulate change (e.g., identify scenarios with greatest efficiency) • Promote adaptability (e.g., tweak planning and supply chains to accommodate new processes)
	PPE	How can we ensure that every health worker has access to, and appropriately dons, PPE?	Adoption, acceptability, fidelity	Stakeholder engagement, social marketing and education (9, 10)	• Develop and distribute educational materials (e.g., social media videos of hand washing techniques, open source patterns for crafting masks on public health websites) • Role revision (e.g., role designated to distribute PPE and ensure appropriate donning)
	Intensive care management	How can we rapidly and efficiently deploy a massive volunteer health force (e.g., doctors and nurses in NYC, or a public health corps)?	Feasibility, penetration	Process mapping, stakeholder engagement, optimization modeling (11, 12), policy analysis (13)	• Role revision and liability laws (e.g., legislation to allow non-ICU health care workers to serve in certain capacities in times of pandemic)
	Intensive care management	How can we ensure that BIPOC communities are receiving equitable screening, testing, and intensive care management? How do we proactively monitor whether tailored responses are equitable for marginalized communities and change our approaches when data reveals inequitable responses?	Adoption, fidelity	Surveillance and data systems (14), policy analysis, quality improvement (15), stakeholder engagement	• Educational meetings and outreach visits (e.g., educating health workers on root causes of inequity, identifying, and rectifying implicit biases) • Involve patients and family members, obtain and use patient and family feedback (e.g., prioritize solutions in partnership with affected communities) • Change service sites (e.g., neighborhoods in which testing and services are available) • Alter patient fee structure (e.g., revise insurance system) • Rapid iterative tests of change, purposely re-examine the implementation, audit and feedback (e.g., disaggregate data sources by race to enable assessments of inequity and track whether interventions are having impact)
	Intensive care management	How can we meet the infrastructure needs required to accommodate patient surges?	Cost, feasibility, penetration	Economic evaluation, optimization modeling	• Change physical structure or equipment (e.g., shift elective areas of hospital into COVID-19 specific wards, convert stadiums and tents into hospitals)
Acting now: enhancing adoption, fidelity, and sustainment of behavioral public health interventions	Social distancing	What kinds of approaches are most effective for increasing fidelity to social distancing? How do we measure and use context to improve transferability of early learnings?	Fidelity	Social marketing, rapid ethnography to understand implementation contexts (16, 17)	• Identify early adopters, inform local leaders, Mandate change (e.g., regional policies) • Alter incentives (e.g., use technology to monitor mobility and integrate individual incentives for high fidelity)
	Social distancing	Are there small tweaks to social distancing policies that improve their effectiveness by making it easier to comply with and sustain?	Acceptability, fidelity, sustainability	Policy analysis, rapid cycle quality improvement	• Promote adaptability and tailor strategies (e.g., retain core element of 6 feet apart distance, but tailor guidance to allow walking 6 feet apart in pairs)
	Social distancing	How does community fidelity to social distancing impact epidemic duration?	Acceptability, fidelity	Qualitative data collection and rapid analysis (18), Network-based models (19), surveillance and data systems	• Use data experts (e.g., utilize passive data, like GPS mobility data aggregated by Google, to inform fidelity near real-time with regional specificity)
	Community behavior change	How does public trust of government officials impact community level compliance with new COVID-19 policies?	Adoption, appropriateness, Fidelity	System dynamics modeling (20), policy analysis	• Educational meetings and outreach visits (e.g., “Town Hall” style meetings with public officials and public health institutions)
	Community behavior change	How can marginalized communities—especially incarcerated or detained persons, persons experiencing homelessness—be enabled to participate in social distancing and other community behavior change activities?	Adoption, appropriateness, fidelity	Policy analysis, stakeholder engagement	• Change physical structure or equipment (e.g., provide free and stable housing, release incarcerated persons from prisons and jails and detention centers) • Mandate change (e.g., regional level executive orders to enable administrative release, legislative change to allow for release during times of pandemic) • Involve patients and family members, obtain and use patient and family feedback (e.g., prioritize solutions in partnership with affected communities through establishment of advisory boards) • Alter incentive structures (e.g., when release is not possible, remove punitive actions associated with quarantine by allowing access to phone, email, etc.) • Role revision (e.g., when release is not possible, retain smaller skeleton staffing that lives on prison campus to minimize external exposure)
	Hand washing and Mask wearing in public spaces	How can we increase coverage of non-medical mask usage in public spaces? Which communication strategies regarding proper hand washing and mask use result in high accuracy and behavioral maintenance on the individual level?	Acceptability, adoption, fidelity, sustainability	Social marketing	• Use mass media, Start a dissemination organization, Develop and distribute educational materials (e.g., social media videos of hand washing techniques, open source patterns for crafting masks on public health websites)
	Cross-cutting	How do we prioritize which policies to deploy (e.g., improve social distancing, mask usage, contact tracing and quarantine, virologic testing coverage, etc.)? How do these decisions change for a resource-limited setting?	Cost, adoption, penetration, sustainability	Simulation models (21), cost-effectiveness analysis, budget impact analysis (22)	• Conduct local needs assessment (e.g., differentiate policies that are engendering targeted behavior changes, by region)
Moving forward: integrating new evidence into decision-making and programming	Testing	How can laboratory testing networks be optimally designed to receive samples within a geographic network and quickly share results back to providers and individuals? How can we ensure that turnaround time is not only fast, but equitable in reaching the multiple, diverse communities who are marginalized?	Appropriateness (practicability), feasibility	Simulation modeling (e.g., queuing, discrete event simulation), surveillance and data systems	• Model and simulate change, change service sites (e.g., consider hub-and-spoke model, disaggregate data sources and model outputs to enable assessments of inequity and prioritize equity in modeled scenarios) • Develop a formal implementation blueprint, Develop and organize quality monitoring systems (e.g., continue to assess test turnaround time and receipt) • Facilitate relay of clinical data to providers (e.g., automated portal for test results sharing, consider sharing directly with patients using portal or text messages) • Use data experts (e.g., outsource creation and management of portal system and automation)
	Testing	What is the most efficient approach to conducting drive-through sample collection for testing? What structural tweaks can be made to this infrastructure to equitably serve populations with diversity in neighborhood, income, wealth, and access to cars?	Appropriateness (practicability), feasibility	Operations research (23), flow mapping, quality improvement, discrete event simulation modeling, optimization modeling	• Rapid iterative tests of change, stage implementation scale up, purposely re-examine the implementation, audit and feedback, develop and organize quality monitoring systems (e.g., continue to assess time spent waiting for sample collection, turnaround time, flow of cars through system and bottlenecks, assess metrics disaggregated by neighborhood and socio-economic status and optimize to ensure equity for each group) • Local knowledge sharing networks, promote network weaving, visit other sites, use train-the-trainer strategies, provide ongoing consultation (e.g., connect new sites with existing expert sites for troubleshooting)
	Testing	How can we overcome structural barriers to testing at the individual and provider levels? How are structural barriers magnified for marginalized communities, including BIPOC, undocumented persons, incarcerated persons, and persons experiencing homelessness? How can structural barriers be mitigated for diverse communities?	Adoption	Policy analysis, costing and cost-effectiveness, Social marketing	• Alter patient fee structure (e.g., insurance, copays, etc.) • Place innovation on fee for service list (e.g., legislation to reimburse telehealth visits at the same rate) • Change service sites (e.g., drive through testing, home-based testing) • Involve patients and family members, obtain and use patient and family feedback (e.g., prioritize solutions in partnership with affected communities)
	Testing	How can clinicians know who to test and how often in a setting of rapidly evolving guidance on test eligibility?	Adoption, fidelity	Policy analysis and leadership engagement, quality improvement	• Remind clinicians and use data warehousing techniques (e.g., integrate symptom check and relevant questions in integrated EMR with automatic flag for test eligibility and prompt to order, harmonize EMR platforms across hospital networks) • Provide local technical assistance, centralize technical assistance (e.g., phone call in line at state public health level for clinicians or county-level public health to clarify protocols)
	Case contact tracing	How can we staff the aggressively high coverage of contact tracing required to relax social distancing policies within a geographic area?	Cost, feasibility, penetration	Cost-effectiveness analysis, policy analysis, Stakeholder mapping and engagement	• Shadow other experts, revise professional roles (e.g., allow expanded lay workforce with training to do contact tracing)
	Case contact tracing	How can a public health response acknowledge the historic injustices that leave diverse marginalized communities differently and disproportionately affected, and adapt their intensive contact tracing approach to be compatible with varied trust in public systems and concerns about privacy?	Acceptability	Social marketing, qualitative data, dissemination science (24, 25)	• Intervene with patients to enhance uptake and adherence, involve patients and family members, Obtain and use patient and family feedback, prepare patients to be active participants, local consensus discussions (e.g., develop communication strategies in partnership with patient populations) • Use mass media, start a dissemination organization • Develop and distribute educational materials
	Future vaccine	In the case of initially limited vaccine availability, how do we prioritize and operationalize vaccination for the most vulnerable? How does that approach and system shift as supplies become more readily available?	Appropriateness (practicability), feasibility, penetration	System dynamics modeling, stakeholder engagement, process mapping	• Assess for readiness and identify barriers and facilitators, facilitate relay of clinical data to providers (e.g., to ensure that standardized approaches are used to identify and prioritize patients)
	Future medication for prophylaxis or treatment	When efficacious medications have been identified for broad prevention, post-exposure prophylaxis, and case treatment, how do we update guidelines for use and broadly distribute?	Adoption, penetration	Social marketing, qualitative data, dissemination science	• Provide clinical supervision, provide ongoing consultation • Increase demand • Intervene with patients/consumers to enhance uptake and adherence • Place innovation on fee for service lists/formularies
	Reducing usage of medications/interventions that are not evidence-based	How can we inhibit or reverse adoption of value-neutral or negative interventions?	De-implementation, adoption	Social marketing, qualitative data, dissemination science	• Remind clinicians and use data warehousing techniques (e.g., integrate diagnosis and treatment check in EMR with automatic flag for test eligibility and prompt to order) • Alter incentives • Local consensus discussions, engage patients

## Reflecting Backward: Role of Is in Planning for Emergency Preparedness Pre-Pandemic

IS can contribute to pandemic preparedness by improving both robustness and agility of systems, drawing from contributing and relevant disciplines such as industrial engineering, business, political science, and more (3, 4, 27). We suggest that robust systems employ expertise and tools that are fit for purpose, while agile systems are built to be adaptable and flexible in quickly changing conditions. IS provides a systems approach to identify and test implementation strategies that can be used to enhance the robustness and agility of systems through the rapid deployment of evidence-based interventions, when available.

Process maps can be used to outline how human and material resources may be distributed under a variety of scenarios. Process mapping methods originated in the manufacturing industry as a quality improvement tool to improve existing processes, or as a formative pre-implementation planning tool (3, 28). To ensure that maps are representative and operational, consensus on the process maps should be reached in advance and account for the perspectives of multi-level stakeholders with responsibilities ranging from planning to front-line service delivery. In this context, microplanning together with extensive stakeholder mapping can establish avenues for deploying evidence-based interventions, even in the absence of specific interventions themselves. These strategies will inevitably require adaptation to fit the context of the disease and necessitated disease management, but will ideally require revision rather than reinvention, saving valuable time during a pandemic.

For example, public health departments in the United States (U.S.) follow the Incident Command System, part of the National Incident Management System, and have developed emergency preparedness systems and policies for how human and material resources—such as PPE, medical workers, case investigators, data collection and reporting staff—are intended to be distributed in times of emergency (29). However, the early COVID-19 response in the U.S. illuminated challenges in the distribution of key resources, such as PPE. Integrated within process mapping development and stakeholder validation processes, implementation scientists can collaborate with public health planners or emergency preparedness personnel to apply simulation and microplanning tools to determine how much PPE would be required under a variety of epidemiologic scenarios and operational constraints affecting their availability (6, 8). Microplans are localized and detailed assessments and plans for implementation, including the resources, timing, and personnel involved (7, 30). Often referred to as “bottom-up planning,” microplans might, for example, detail PPE requirements and constraints across a variety of federal funding scenarios, reproductive rates, and supply chain structures. Newly developed microplans can, as a result, provide a data-informed and tiered planning protocol where stockpiles are iteratively adjusted as human resources fluctuate or supply chains are compromised due to changes in distribution channels (Table 2). Such an approach requires redefining concepts of “lean” implementation at present to consider future wastage of time, a vital resource during a pandemic. Additional examples of how IS might be applied to planning for emergency preparedness are reflected in Table 2.

## Acting Now: Enhancing Adoption, Fidelity, and Sustainment of Hand Washing and Social Distancing

In the absence of widespread testing, bending the COVID-19 curve is predicated on broad uptake of social distancing and individual-level hygiene behaviors. Public health departments and medical institutions throughout the world have invested in health communication and social mobilization to communicate the importance of these activities with communities. Linking qualitative data (including data from the private sector, such as Google searches, YouTube views, and GPS mobility), policy analysis, and dissemination science can produce real-time information regarding public reactions to government and employer-issued guidance, which can be used to understand what social marketing strategies penetrate effectively with target populations. These insights can inform quick revision of social marketing strategies and policy design in other affected jurisdictions with compatible implementation climates. For example, lessons learned from early messaging might be transferable between settings with comparable population demographics, health care infrastructure, and public health departments to increase message penetration earlier in an epidemic. Similarly, incorporating rapid feedback from stakeholders is necessary for ensuring that programs perform as expected (Table 2). Rapid feedback from community members can be collated during an outbreak using social media, crowdsourced innovations, or media monitoring. Rapid feedback from clinical or public health responders can be collated by appointing an information disseminator, whose primary responsibility is to attend daily planning/debriefing meetings to document what activities are working well, and which require adaptation or abandonment. These lessons learned can then be disseminated to community members and other health jurisdictions periodically.

Different populations have differential ability to adopt and sustain social distancing interventions; immediate IS actions must explicitly take an equity lens (31). For example, social distancing is not feasible for incarcerated individuals or undocumented individuals in detention (32). Orders to “stay home, stay healthy” are not possible for individuals experiencing homelessness (33). COVID-19 has disproportionately affected Black, indigenous, and persons of color (BIPOC) communities; guidelines to seek testing and medical management are differentially experienced by communities of color who may have poorer access to services and higher risk of poor health outcomes from COVID-19 due to historic and current injustices, individual and structural racism, environmental racism, and bias in the quality of health services delivered (34). Employing IS methods and strategies that illuminate and combat inequity—such as disaggregating surveillance data by race, changing physical structures to provide housing and to release detained or incarcerated persons, altering incentive structures to make testing and medical coverage independent of citizenship, engaging communities in identifying and testing solutions—will be essential for immediate impact on equity (Table 2). However, mitigating the impacts of inequity at an individual and organizational level is insufficient; a simultaneous approach to address upstream drivers of inequity and dismantle oppressive systems at the structural level is essential for future public health prevention. Additional examples of how IS can be utilized now to curb epidemic spread are presented in Table 2.

A key implementation determinant influencing adoption, fidelity, and cost of COVID-19 control strategies is ineffective communication channels, resulting in protocols that are interpreted differently across settings and unnecessary implementation delays. For example, a large number of health workers have been infected, resulting in a health workforce that is stretched thinly in many areas (35, 36). Yet, in the U.S., confusion regarding when health workers can return to work after symptom resolution has exacerbated this problem due to existence of multiple protocols (37). Delays clarifying return to work protocols at one point appeared tied to faulty supply of testing kits, rather than evidence (Table 1) (38).

## Moving Forward: Integrating New Evidence Into Decision-Making

Even in the absence of an evidence-based intervention, IS can guide evidence-based decision-making as data become available. This means having systems that help governments and health facilities incorporate evidence agilely: key attributes include strengthening stakeholder networks with clear communication channels, social mobilization mechanisms that can be quickly activated, and microplanning tools to rapidly guide resource allocation. As we transition to recovery and reflection from the pandemic, key implementation outcomes to consider include penetration and sustainability (Table 1). For penetration, we ask whether best practices of microplanning and stakeholder engagement have been integrated into routine pandemic preparedness in all countries and health jurisdictions? For sustainability, we evaluate whether governments have established necessary policies and programs needed to support these activities longer-term, throughout recovery, and evolving back into preparedness. Key implementation science questions related to enhancing these implementation outcomes applied to current and future evidence based interventions are highlighted in Table 2.

Advanced use of modeling aimed at pragmatic decision-making can inform efficient resource allocation—human resources, tests, PPE, ventilators—when they become available, avoiding bottlenecks in distribution seen with volunteer medical personnel in New York City (39). Sophisticated mathematical models ideally suited to answer epidemiologic spread questions have already been useful in informing decision-making; these complex models are resource-intensive to create and are best suited to scenarios where existing models can be adapted, or a new model can inform many “what if” scenarios (40–43). Sophisticated mathematical models have been crucial in informing how conditions would need to be modified to not overload an existing health system, which has influenced state-level policies on social distancing and shelter in place policy timing (41). Additionally, this modeling has been translated into accessible infographics to reinforce the immediate urgency of fidelity to social distancing to curb spread.

In contrast, several types of systems engineering models may be less resource intensive to create and can be useful to inform local decision-making (Table 2). For example, as serologic tests are developed and virologic testing capacity expands, there are questions of where to situate central laboratories within hub-and-spoke models or how to arrange distribution of rapid point-of-care tests from a centralized point. For example, queuing modeling has been used to prospectively plan for resource allocation for other diseases, such as HIV (21, 44). As an effective vaccine becomes available, advanced use of these models can accelerate efficient distribution by prioritizing certain values—such as maximizing equity, or minimizing costs—and can compare a range of scenarios for decision-makers. These modeling tools can be developed now, as new evidence-based interventions are either created or scaled up.

## Learning About Implementation During COVID-19

IS is an emerging discipline with rapidly evolving methods for measuring outcomes and determinants, testing strategies, and assessing context; as with any emerging discipline, it takes time to develop both rigorous and pragmatic methods. However, time invested in measurement calibration can contribute to a further delay in getting “what works,” evidence-based interventions, to populations who need them. The COVID-19 pandemic makes a critical case for rapid, rigorous, nimble, and pragmatic IS.

The pandemic response highlights two lacunae in IS foci. First, an inattention to supply chain and logistics. IS conducted in low-and-middle income countries often addresses resource supply and logistics because they are acutely and chronically observed as implementation determinants (45, 46). Yet, they are relatively absent from IS literature in high-income countries. A second gap is inattention to politics as an implementation barrier or facilitator. While external policies and incentives are addressed in many established frameworks and checklists, implementation scientists do not often incorporate political science perspectives or theories within their work (47, 48). Many of the suggestions presented in this perspective depend upon and assume government cooperation with health departments and accountability to scientific leadership. The COVID-19 pandemic has made it abundantly clear that implementation scientists can no longer overlook the importance of politics as a major implementation determinant.

In the immediate future, implementation scientists can be more “real time” with their questions and findings by using routinely available data, rather than primary data; for example, aggregated trends in Google mobility data as a proxy for fidelity to social distancing. Implementation scientists can utilize non-experimental designs for robust impact evaluation—such as regression discontinuity (including interrupted time series) and difference in differences—without additional dedicated research funding. In the coming years, IS as a discipline can focus on improving the rigor and rapidity of validated measures—including simple scales for assessing implementation outcomes, determinants, and context. These measures should be relevant across geographic and sociocultural settings. Finally, IS will benefit from more purposeful integration within public health departments and health organizations, not exclusively within academic organizations.

## Conclusions

Many of the challenges hindering effective COVID-19 response to date have been driven by operational, logistical, and social factors. IS, public health, medical, and governmental communities can work together to inform a stronger response to the COVID-19 pandemic. Missed opportunities in the past illuminate opportunities for future revisions in how we approach emergency preparedness planning. Simultaneously, this pandemic challenges implementation scientists to continue to endeavor to become more rapid and nimble in addressing the public health emergencies of the future.

## Data Availability Statement

The original contributions presented in the study are included in the article/supplementary material, further inquiries can be directed to the corresponding author/s.

## Author Contributions

AW and AM conceptualized and wrote the first draft of the manuscript. EK, LN, and BW reviewed and revised the manuscript. All authors reviewed and approved the final version of this manuscript.

## Conflict of Interest

The authors declare that the research was conducted in the absence of any commercial or financial relationships that could be construed as a potential conflict of interest.
